# Synergy between liposomal zoledronic acid and γδ T-cells in the treatment of epithelial ovarian cancer

**DOI:** 10.1186/2051-1426-1-S1-P28

**Published:** 2013-11-07

**Authors:** Ana Catarina Parente Pereira, Hilary Shmeeda, Lynsey Whilding, Sadaf Ghaem-Maghami, Alberto Gabizon, John Maher

**Affiliations:** 1 Kings College London, London, UK; 2 Shaare Zedek Medical Center, Jerusalem, Israel; 3 Imperial College London, London, UK

## 

γδ T-cells contribute importantly to tumor immunosurveillance and are activated by phosphoantigen intermediates of the mevalonate pathway that are commonly over-produced in cancer cells. To potentiate this, phosphoantigen levels can be boosted using zoledronic acid (ZA). However, in vivo delivery of ZA to the tumour is inefficient owing to its poor pharmacokinetic properties. Here, we set out to develop a γδ T-cell immunotherapy for epithelial ovarian cancer (EOC) using folate-targeted liposomal ZA (FT-L-ZA) to improve drug delivery to tumor cells. Folate receptor-α is over-expressed in EOC. Peripheral blood mononuclear cells isolated from healthy donors (n=21) and EOC patients (n=13) were cultured with ZA, IL-2 and IL-15 for two weeks. γδ T-cells expanded reproducibly from healthy donors and patients, the latter having on average a 97-fold expansion. The expanded γδ T-cells expressed low levels of L-selectin, accompanied by CD45RO, CD27, CD70 and NKG2D, consistent with a combined central-memory and effector-memory phenotype, similarly to healthy controls. To evaluate whether ex-vivo cultured γδ T-cells from patients with EOC are functionally competent, we developed an autologous co-cultivation assay whereby patient-derived γδ T-cells were incubated in-vitro with primary EOC “tumorspheres”. Twenty-four hours after incubation of tumorspheres with ZA, L-ZA or FT-L-ZA, ex-vivo expanded γδ T-cells were added at a 1:100 ratio. When alone, γδ T-cells destroyed 24.5% of spheres. Addition of 1µg/ml free ZA sensitized tumorspheres to destruction by γδ T-cells (96.2%), but was relatively ineffective at 0.1µg/ml (26.8%). By contrast, FT-L-ZA was highly effective at 0.1µg/ml (92.6%) while non-targeted L-ZA was no better than γδ T-cells only. Destruction of ZA-sensitized tumorspheres by autologous γδ T-cells was accompanied by their activation and production IFN−γ (≈ 2000 pg/mL). In the absence of γδ T-cells, none of these formulations exerted any toxic effect upon tumorspheres. To model EOC in vivo, SCID Beige mice were injected intraperitoneally (IP) with luciferase-expressing SKOV-3 cells. Mice (n=5 per group) were treated IP with 5μg ZA, FT-L-ZA or PBS, followed by 10E7 ex-vivo expanded γδ T-cells from healthy controls or PBS. Tumour growth was monitored by bioluminescence. One week after treatment, mice treated with γδ T-cells or γδ T-cells and free ZA showed a tumour reduction of approximately 20%. By contrast, mice treated with FT-L-ZA followed by γδ T-cells achieved a tumour reduction of 60-70%. Drugs alone had no therapeutic effect. We show here proof of concept for a synergistic immunotherapy for EOC whereby FT-L-ZA can be used to sensitize tumors to γδ T-cells.

